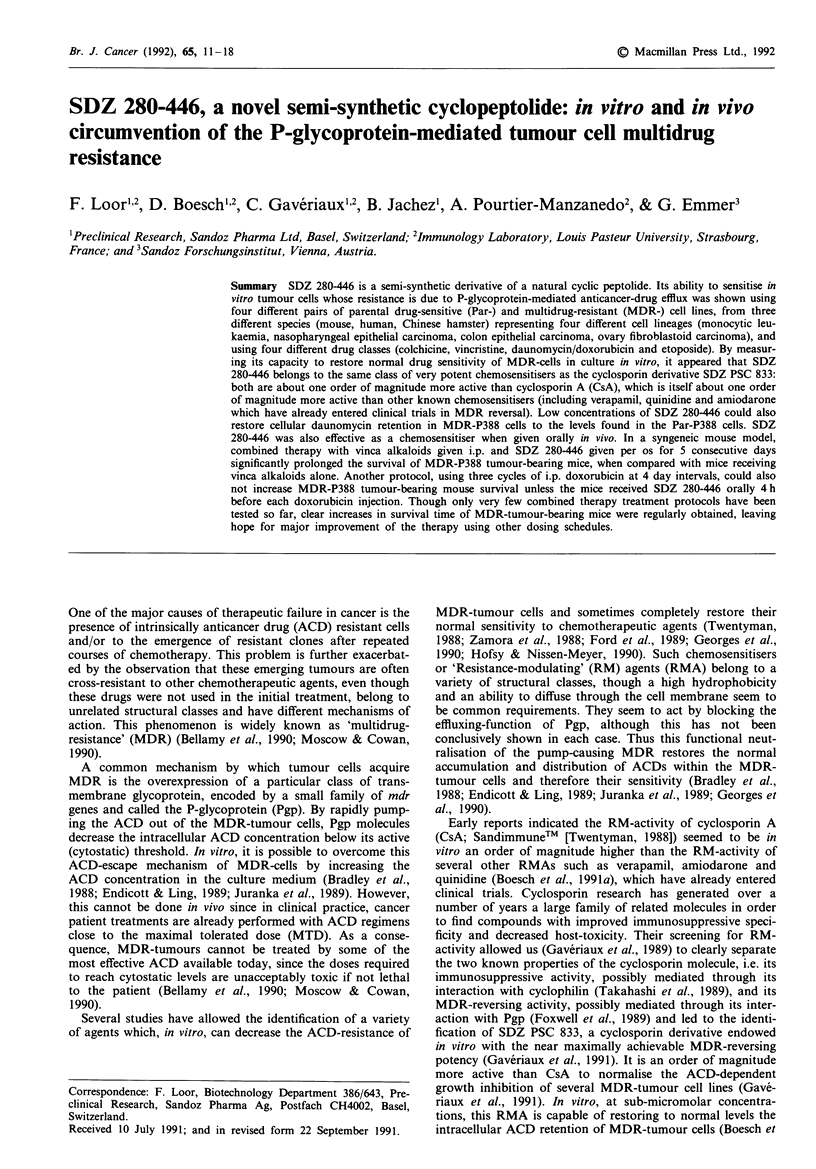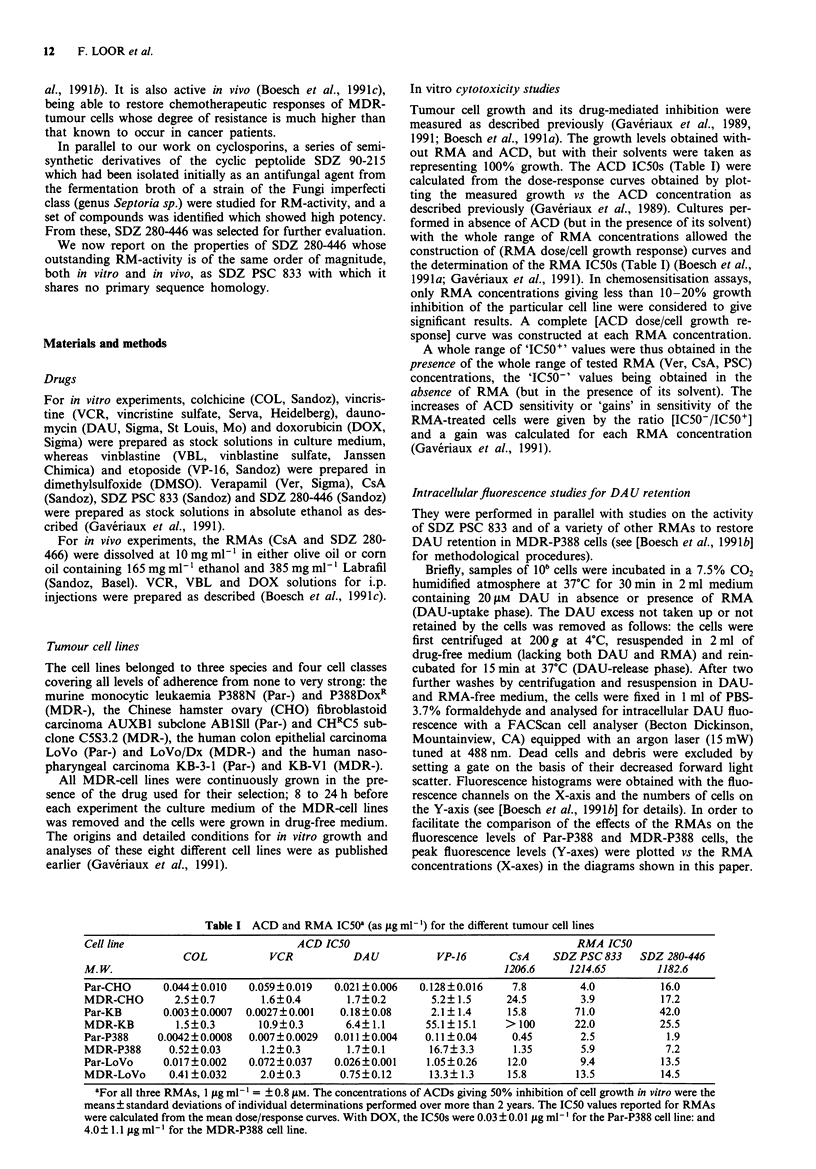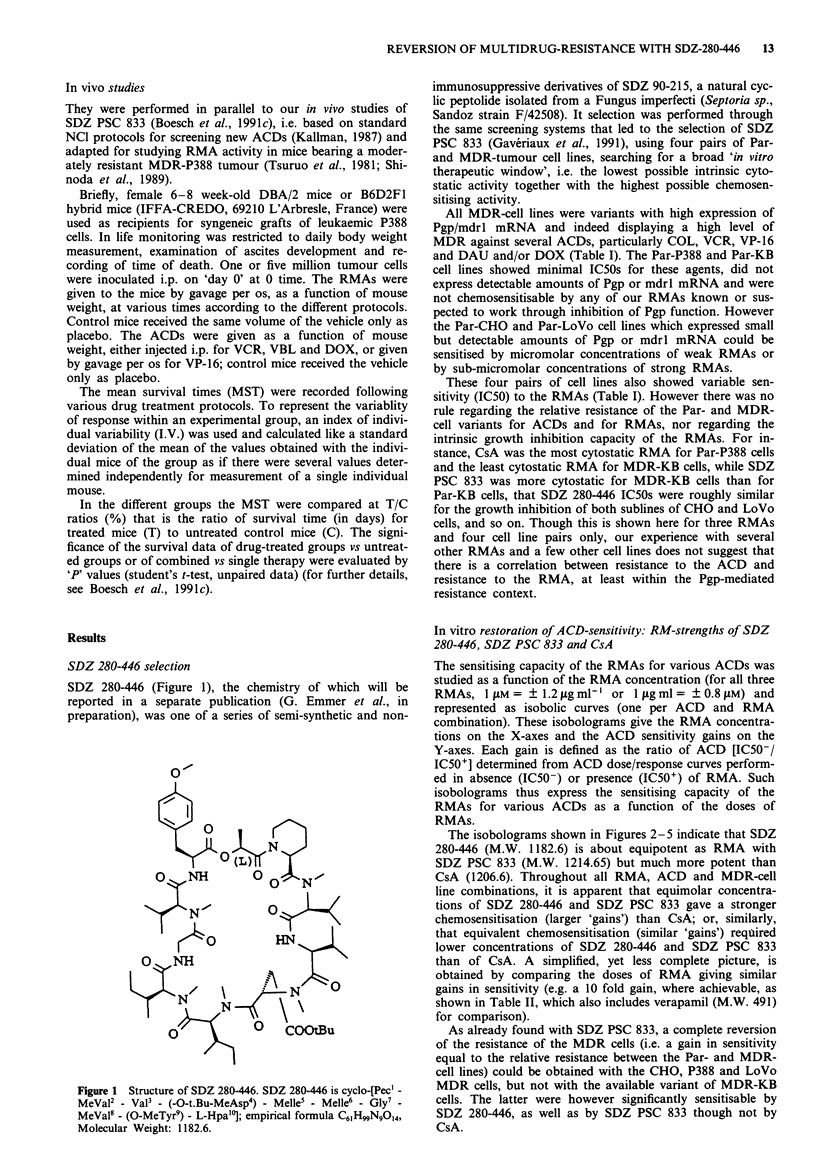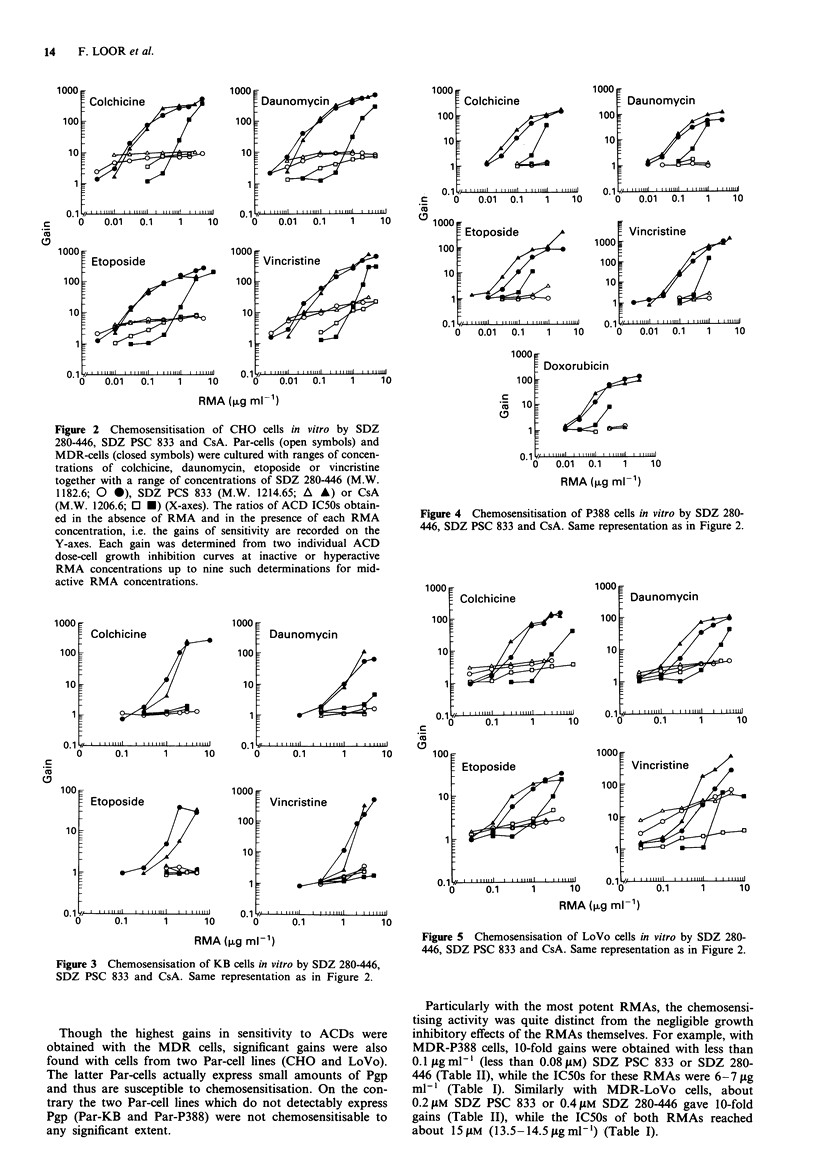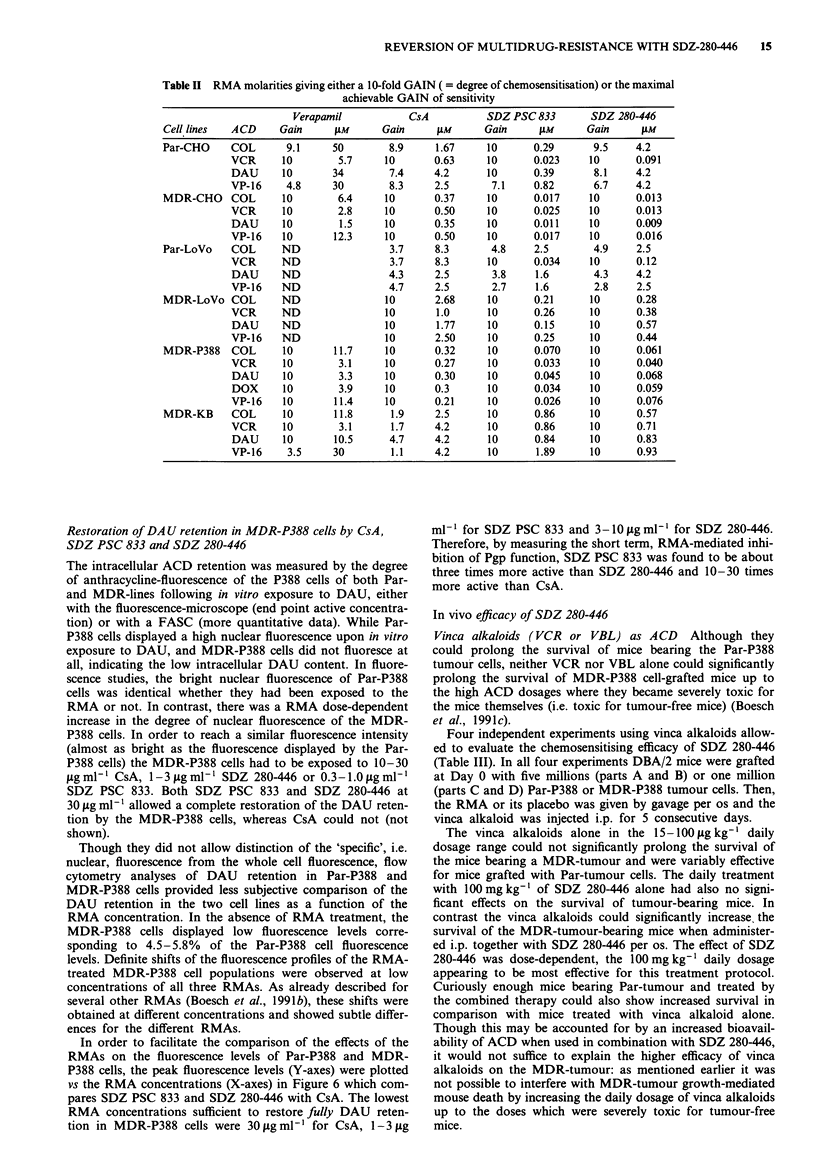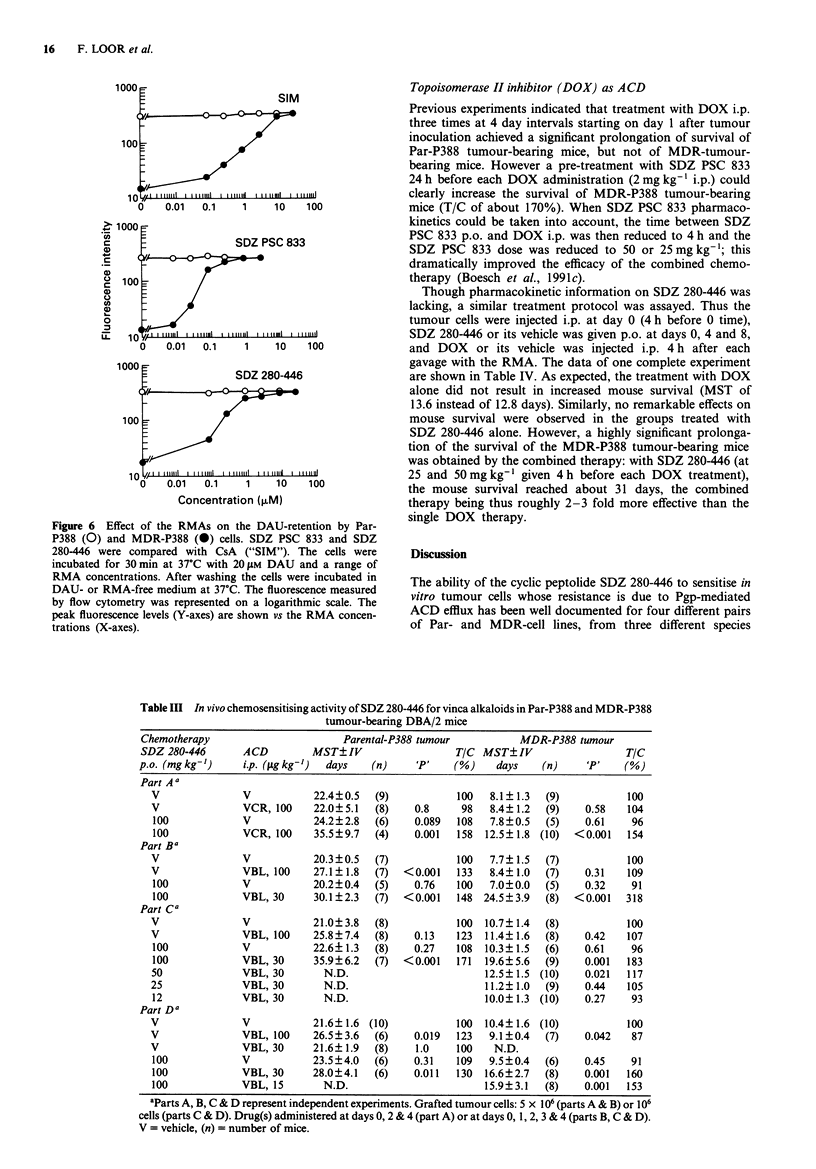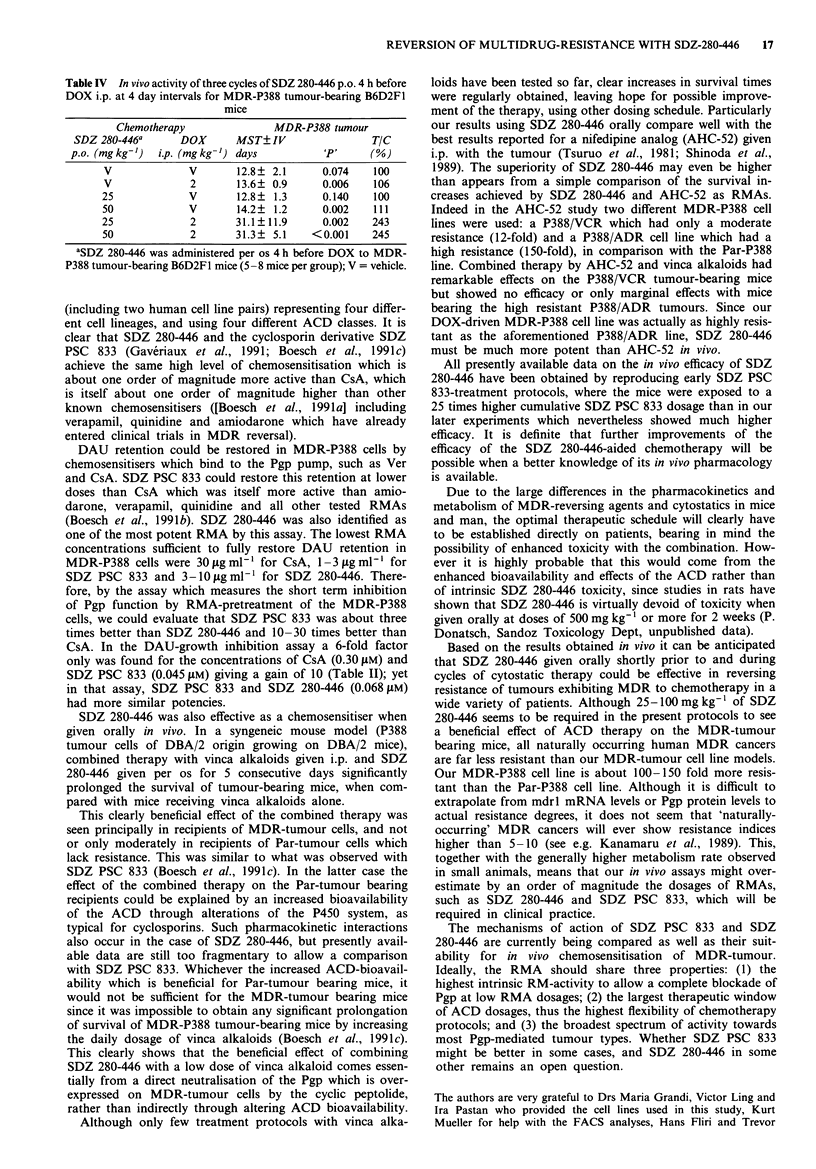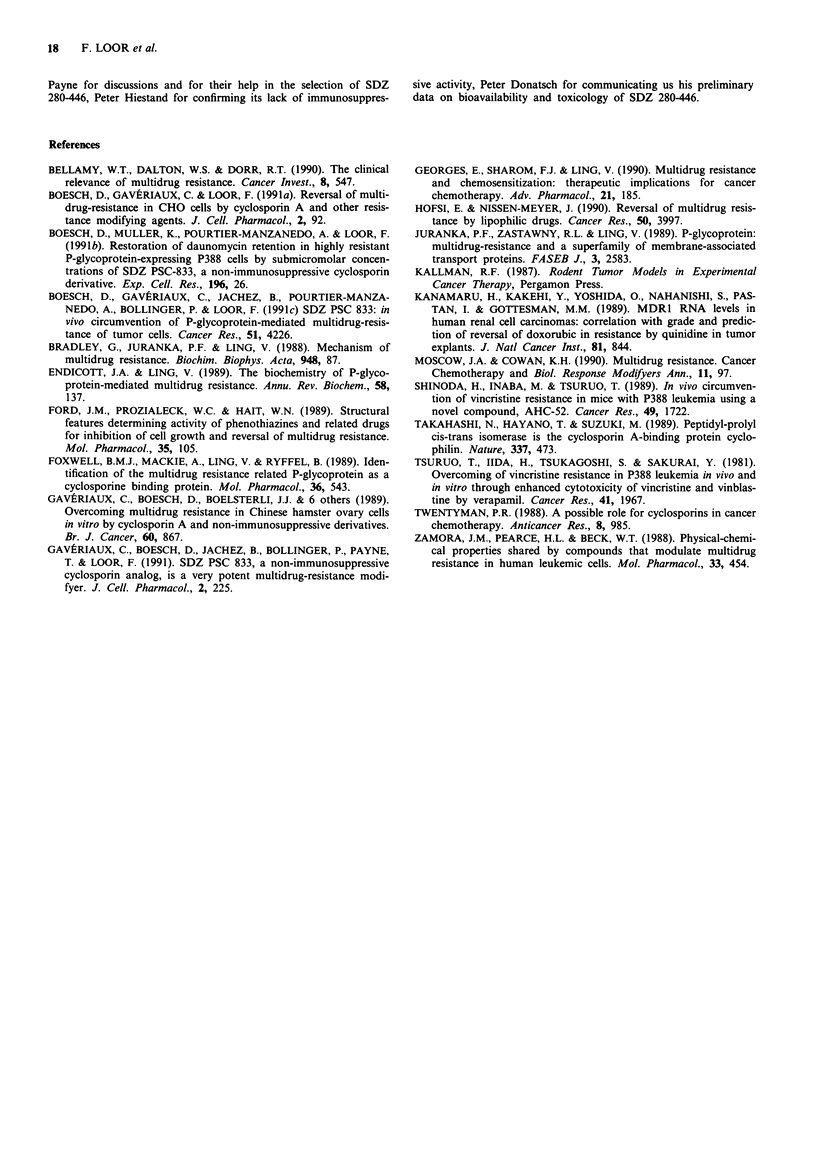# SDZ 280-446, a novel semi-synthetic cyclopeptolide: in vitro and in vivo circumvention of the P-glycoprotein-mediated tumour cell multidrug resistance.

**DOI:** 10.1038/bjc.1992.3

**Published:** 1992-01

**Authors:** F. Loor, D. Boesch, C. Gavériaux, B. Jachez, A. Pourtier-Manzanedo, G. Emmer

**Affiliations:** Biotechnology Department, Sandoz Pharma Ltd, Basel, Switzerland.

## Abstract

SDZ 280-446 is a semi-synthetic derivative of a natural cyclic peptolide. Its ability to sensitise in vitro tumour cells whose resistance is due to P-glycoprotein-mediated anticancer-drug efflux was shown using four different pairs of parental drug-sensitive (Par-) and multidrug-resistant (MDR-) cell lines, from three different species (mouse, human, Chinese hamster) representing four different cell lineages (monocytic leukaemia, nasopharyngeal epithelial carcinoma, colon epithelial carcinoma, ovary fibroblastoid carcinoma), and using four different drug classes (colchicine, vincristine, daunomycin/doxorubicin and etoposide). By measuring its capacity to restore normal drug sensitivity of MDR-cells in culture in vitro, it appeared that SDZ 280-446 belongs to the same class of very potent chemosensitisers as the cyclosporin derivative SDZ PSC 833: both are about one order of magnitude more active than cyclosporin A (CsA), which is itself about one order of magnitude more active than other known chemosensitisers (including verapamil, quinidine and amiodarone which have already entered clinical trials in MDR reversal). Low concentrations of SDZ 280-446 could also restore cellular daunomycin retention in MDR-P388 cells to the levels found in the Par-P388 cells. SDZ 280-446 was also effective as a chemosensitiser when given orally in vivo. In a syngeneic mouse model, combined therapy with vinca alkaloids given i.p. and SDZ 280-446 given per os for 5 consecutive days significantly prolonged the survival of MDR-P388 tumour-bearing mice, when compared with mice receiving vinca alkaloids alone. Another protocol, using three cycles of i.p. doxorubicin at 4 day intervals, could also not increase MDR-P388 tumour-bearing mouse survival unless the mice received SDZ 280-446 orally 4 h before each doxorubicin injection. Though only very few combined therapy treatment protocols have been tested so far, clear increases in survival time of MDR-tumour-bearing mice were regularly obtained, leaving hope for major improvement of the therapy using other dosing schedules.


Br. J. Cancer (1992), 65, 11-18 © Macmillan Press Ltd., 1992

### SDZ 280-446, a novel semi-synthetic cyclopeptolide: *in vitro* and *in vivo* circumvention of the P-glycoprotein-mediated tumour cell multidrug resistance

F. Loor[1,2], D. Boesch[1,2], C. Gavériaux[1,2], B. Jachez[1], A. Pourtier-Manzanedo[2], & G. Emmer[3]

[1]*Preclinical Research, Sandoz Pharma Ltd, Basel, Switzerland;* [2]*Immunology Laboratory, Louis Pasteur University, Strasbourg, France; and* [3]*Sandoz Forschungsinstitut, Vienna, Austria.*

**Summary** SDZ 280-446 is a semi-synthetic derivative of a natural cyclic peptolide. Its ability to sensitise *in vitro* tumour cells whose resistance is due to P-glycoprotein-mediated anticancer-drug efflux was shown using four different pairs of parental drug-sensitive (Par-) and multidrug-resistant (MDR-) cell lines, from three different species (mouse, human, Chinese hamster) representing four different cell lineages (monocytic leukaemia, nasopharyngeal epithelial carcinoma, colon epithelial carcinoma, ovary fibroblastoid carcinoma), and using four different drug classes (colchicine, vincristine, daunomycin/doxorubicin and etoposide). By measuring its capacity to restore normal drug sensitivity of MDR-cells in culture *in vitro*, it appeared that SDZ 280-446 belongs to the same class of very potent chemosensitisers as the cyclosporin derivative SDZ PSC 833: both are about one order of magnitude more active than cyclosporin A (CsA), which is itself about one order of magnitude more active than other known chemosensitisers (including verapamil, quinidine and amiodarone which have already entered clinical trials in MDR reversal). Low concentrations of SDZ 280-446 could also restore cellular daunomycin retention in MDR-P388 cells to the levels found in the Par-P388 cells. SDZ 280-446 was also effective as a chemosensitiser when given orally *in vivo*. In a syngeneic mouse model, combined therapy with vinca alkaloids given i.p. and SDZ 280-446 given per os for 5 consecutive days significantly prolonged the survival of MDR-P388 tumour-bearing mice, when compared with mice receiving vinca alkaloids alone. Another protocol, using three cycles of i.p. doxorubicin at 4 day intervals, could also not increase MDR-P388 tumour-bearing mouse survival unless the mice received SDZ 280-446 orally 4 h before each doxorubicin injection. Though only very few combined therapy treatment protocols have been tested so far, clear increases in survival time of MDR-tumour-bearing mice were regularly obtained, leaving hope for major improvement of the therapy using other dosing schedules.

One of the major causes of therapeutic failure in cancer is the presence of intrinsically anticancer drug (ACD) resistant cells and/or to the emergence of resistant clones after repeated courses of chemotherapy. This problem is further exacerbated by the observation that these emerging tumours are often cross-resistant to other chemotherapeutic agents, even though these drugs were not used in the initial treatment, belong to unrelated structural classes and have different mechanisms of action. This phenomenon is widely known as 'multidrug-resistance' (MDR) (Bellamy *et al.*, 1990; Moscow & Cowan, 1990).

A common mechanism by which tumour cells acquire MDR is the overexpression of a particular class of transmembrane glycoprotein, encoded by a small family of *mdr* genes and called the P-glycoprotein (Pgp). By rapidly pumping the ACD out of the MDR-tumour cells, Pgp molecules decrease the intracellular ACD concentration below its active (cytostatic) threshold. *In vitro*, it is possible to overcome this ACD-escape mechanism of MDR-cells by increasing the ACD concentration in the culture medium (Bradley *et al.*, 1988; Endicott & Ling, 1989; Juranka *et al.*, 1989). However, this cannot be done *in vivo* since in clinical practice, cancer patient treatments are already performed with ACD regimens close to the maximal tolerated dose (MTD). As a consequence, MDR-tumours cannot be treated by some of the most effective ACD available today, since the doses required to reach cytostatic levels are unacceptably toxic if not lethal to the patient (Bellamy *et al.*, 1990; Moscow & Cowan, 1990).

Several studies have allowed the identification of a variety of agents which, *in vitro*, can decrease the ACD-resistance of MDR-tumour cells and sometimes completely restore their normal sensitivity to chemotherapeutic agents (Twentyman, 1988; Zamora *et al.*, 1988; Ford *et al.*, 1989; Georges *et al.*, 1990; Hofsy & Nissen-Meyer, 1990). Such chemosensitisers or 'Resistance-modulating' (RM) agents (RMA) belong to a variety of structural classes, though a high hydrophobicity and an ability to diffuse through the cell membrane seem to be common requirements. They seem to act by blocking the effluxing-function of Pgp, although this has not been conclusively shown in each case. Thus this functional neutralisation of the pump-causing MDR restores the normal accumulation and distribution of ACDs within the MDR-tumour cells and therefore their sensitivity (Bradley *et al.*, 1988; Endicott & Ling, 1989; Juranka *et al.*, 1989; Georges *et al.*, 1990).

Early reports indicated the RM-activity of cyclosporin A (CsA; Sandimmune™ [Twentyman, 1988]) seemed to be *in vitro* an order of magnitude higher than the RM-activity of several other RMAs such as verapamil, amiodarone and quinidine (Boesch *et al.*, 1991*a*), which have already entered clinical trials. Cyclosporin research has generated over a number of years a large family of related molecules in order to find compounds with improved immunosuppressive specificity and decreased host-toxicity. Their screening for RM-activity allowed us (Gavériaux *et al.*, 1989) to clearly separate the two known properties of the cyclosporin molecule, i.e. its immunosuppressive activity, possibly mediated through its interaction with cyclophilin (Takahashi *et al.*, 1989), and its MDR-reversing activity, possibly mediated through its interaction with Pgp (Foxwell *et al.*, 1989) and led to the identification of SDZ PSC 833, a cyclosporin derivative endowed *in vitro* with the near maximally achievable MDR-reversing potency (Gavériaux *et al.*, 1991). It is an order of magnitude more active than CsA to normalise the ACD-dependent growth inhibition of several MDR-tumour cell lines (Gavériaux *et al.*, 1991). *In vitro*, at sub-micromolar concentrations, this RMA is capable of restoring to normal levels the intracellular ACD retention of MDR-tumour cells (Boesch *et*

Correspondence: F. Loor, Biotechnology Department 386/643, Preclinical Research, Sandoz Pharma Ag, Postfach CH4002, Basel, Switzerland.
Received 10 July 1991; and in revised form 22 September 1991.

al., 1991b). It is also active *in vivo* (Boesch *et al.*, 1991c), being able to restore chemotherapeutic responses of MDR-tumour cells whose degree of resistance is much higher than that known to occur in cancer patients.

In parallel to our work on cyclosporins, a series of semi-synthetic derivatives of the cyclic peptolide SDZ 90-215 which had been isolated initially as an antifungal agent from the fermentation broth of a strain of the Fungi imperfecti class (genus *Septoria sp.*) were studied for RM-activity, and a set of compounds was identified which showed high potency. From these, SDZ 280-446 was selected for further evaluation.

We now report on the properties of SDZ 280-446 whose outstanding RM-activity is of the same order of magnitude, both *in vitro* and *in vivo*, as SDZ PSC 833 with which it shares no primary sequence homology.

### Materials and methods

### *Drugs*

For *in vitro* experiments, colchicine (COL, Sandoz), vincristine (VCR, vincristine sulfate, Serva, Heidelberg), daunomycin (DAU, Sigma, St Louis, Mo) and doxorubicin (DOX, Sigma) were prepared as stock solutions in culture medium, whereas vinblastine (VBL, vinblastine sulfate, Janssen Chimica) and etoposide (VP-16, Sandoz) were prepared in dimethylsulfoxide (DMSO). Verapamil (Ver, Sigma), CsA (Sandoz), SDZ PSC 833 (Sandoz) and SDZ 280-446 (Sandoz) were prepared as stock solutions in absolute ethanol as described (Gavériaux *et al.*, 1991).

For *in vivo* experiments, the RMAs (CsA and SDZ 280-466) were dissolved at 10 mg $ml^{-1}$ in either olive oil or corn oil containing 165 mg $ml^{-1}$ ethanol and 385 mg $ml^{-1}$ Labrafil (Sandoz, Basel). VCR, VBL and DOX solutions for i.p. injections were prepared as described (Boesch *et al.*, 1991c).

### *Tumour cell lines*

The cell lines belonged to three species and four cell classes covering all levels of adherence from none to very strong: the murine monocytic leukaemia P388N (Par-) and P388Dox$^R$ (MDR-), the Chinese hamster ovary (CHO) fibroblastoid carcinoma AUXB1 subclone AB1S11 (Par-) and CH$^R$C5 subclone C5S3.2 (MDR-), the human colon epithelial carcinoma LoVo (Par-) and LoVo/Dx (MDR-) and the human nasopharyngeal carcinoma KB-3-1 (Par-) and KB-V1 (MDR-).

All MDR-cell lines were continuously grown in the presence of the drug used for their selection; 8 to 24 h before each experiment the culture medium of the MDR-cell lines was removed and the cells were grown in drug-free medium. The origins and detailed conditions for *in vitro* growth and analyses of these eight different cell lines were as published earlier (Gavériaux *et al.*, 1991).

### In vitro *cytotoxicity studies*

Tumour cell growth and its drug-mediated inhibition were measured as described previously (Gavériaux *et al.*, 1989, 1991; Boesch *et al.*, 1991a). The growth levels obtained without RMA and ACD, but with their solvents were taken as representing 100% growth. The ACD IC50s (Table I) were calculated from the dose-response curves obtained by plotting the measured growth *vs* the ACD concentration as described previously (Gavériaux *et al.*, 1989). Cultures performed in absence of ACD (but in the presence of its solvent) with the whole range of RMA concentrations allowed the construction of (RMA dose/cell growth response) curves and the determination of the RMA IC50s (Table I) (Boesch *et al.*, 1991a; Gavériaux *et al.*, 1991). In chemosensitisation assays, only RMA concentrations giving less than 10–20% growth inhibition of the particular cell line were considered to give significant results. A complete [ACD dose/cell growth response] curve was constructed at each RMA concentration.

A whole range of 'IC50$^+$' values were thus obtained in the *presence* of the whole range of tested RMA (Ver, CsA, PSC) concentrations, the 'IC50$^-$' values being obtained in the *absence* of RMA (but in the presence of its solvent). The increases of ACD sensitivity or 'gains' in sensitivity of the RMA-treated cells were given by the ratio [IC50$^-$/IC50$^+$] and a gain was calculated for each RMA concentration (Gavériaux *et al.*, 1991).

### *Intracellular fluorescence studies for DAU retention*

They were performed in parallel with studies on the activity of SDZ PSC 833 and of a variety of other RMAs to restore DAU retention in MDR-P388 cells (see [Boesch *et al.*, 1991b] for methodological procedures).

Briefly, samples of $10^6$ cells were incubated in a 7.5% $CO_2$ humidified atmosphere at 37°C for 30 min in 2 ml medium containing 20 μM DAU in absence or presence of RMA (DAU-uptake phase). The DAU excess not taken up or not retained by the cells was removed as follows: the cells were first centrifuged at 200 *g* at 4°C, resuspended in 2 ml of drug-free medium (lacking both DAU and RMA) and reincubated for 15 min at 37°C (DAU-release phase). After two further washes by centrifugation and resuspension in DAU- and RMA-free medium, the cells were fixed in 1 ml of PBS-3.7% formaldehyde and analysed for intracellular DAU fluorescence with a FACScan cell analyser (Becton Dickinson, Mountainview, CA) equipped with an argon laser (15 mW) tuned at 488 nm. Dead cells and debris were excluded by setting a gate on the basis of their decreased forward light scatter. Fluorescence histograms were obtained with the fluorescence channels on the X-axis and the numbers of cells on the Y-axis (see [Boesch *et al.*, 1991b] for details). In order to facilitate the comparison of the effects of the RMAs on the fluorescence levels of Par-P388 and MDR-P388 cells, the peak fluorescence levels (Y-axes) were plotted *vs* the RMA concentrations (X-axes) in the diagrams shown in this paper.

**Table I** ACD and RMA IC50[a] (as μg $ml^{-1}$) for the different tumour cell lines

| *Cell line* | ACD IC50 | | | | RMA IC50 | | |
|---|---|---|---|---|---|---|---|
| | *COL* | *VCR* | *DAU* | *VP-16* | *CsA* | *SDZ PSC 833* | *SDZ 280-446* |
| *M.W.* | | | | | *1206.6* | *1214.65* | *1182.6* |
| Par-CHO | 0.044±0.010 | 0.059±0.019 | 0.021±0.006 | 0.128±0.016 | 7.8 | 4.0 | 16.0 |
| MDR-CHO | 2.5±0.7 | 1.6±0.4 | 1.7±0.2 | 5.2±1.5 | 24.5 | 3.9 | 17.2 |
| Par-KB | 0.003±0.0007 | 0.0027±0.001 | 0.18±0.08 | 2.1±1.4 | 15.8 | 71.0 | 42.0 |
| MDR-KB | 1.5±0.3 | 10.9±0.3 | 6.4±1.1 | 55.1±15.1 | >100 | 22.0 | 25.5 |
| Par-P388 | 0.0042±0.0008 | 0.007±0.0029 | 0.011±0.004 | 0.11±0.04 | 0.45 | 2.5 | 1.9 |
| MDR-P388 | 0.52±0.03 | 1.2±0.3 | 1.7±0.1 | 16.7±3.3 | 1.35 | 5.9 | 7.2 |
| Par-LoVo | 0.017±0.002 | 0.072±0.037 | 0.026±0.001 | 1.05±0.26 | 12.0 | 9.4 | 13.5 |
| MDR-LoVo | 0.41±0.032 | 2.0±0.3 | 0.75±0.12 | 13.3±1.3 | 15.8 | 13.5 | 14.5 |

[a]For all three RMAs, 1 μg $ml^{-1}$ = ±0.8 μM. The concentrations of ACDs giving 50% inhibition of cell growth *in vitro* were the means±standard deviations of individual determinations performed over more than 2 years. The IC50 values reported for RMAs were calculated from the mean dose/response curves. With DOX, the IC50s were 0.03±0.01 μg $ml^{-1}$ for the Par-P388 cell line: and 4.0±1.1 μg $ml^{-1}$ for the MDR-P388 cell line.

### In vivo *studies*

They were performed in parallel to our *in vivo* studies of SDZ PSC 833 (Boesch *et al*., 1991*c*), i.e. based on standard NCl protocols for screening new ACDs (Kallman, 1987) and adapted for studying RMA activity in mice bearing a moderately resistant MDR-P388 tumour (Tsuruo *et al*., 1981; Shinoda *et al*., 1989).

Briefly, female 6–8 week-old DBA/2 mice or B6D2F1 hybrid mice (IFFA-CREDO, 69210 L'Arbresle, France) were used as recipients for syngeneic grafts of leukaemic P388 cells. In life monitoring was restricted to daily body weight measurement, examination of ascites development and recording of time of death. One or five million tumour cells were inoculated i.p. on 'day 0' at 0 time. The RMAs were given to the mice by gavage per os, as a function of mouse weight, at various times according to the different protocols. Control mice received the same volume of the vehicle only as placebo. The ACDs were given as a function of mouse weight, either injected i.p. for VCR, VBL and DOX, or given by gavage per os for VP-16; control mice received the vehicle only as placebo.

The mean survival times (MST) were recorded following various drug treatment protocols. To represent the variablity of response within an experimental group, an index of individual variability (I.V.) was used and calculated like a standard deviation of the mean of the values obtained with the individual mice of the group as if there were several values determined independently for measurement of a single individual mouse.

In the different groups the MST were compared at T/C ratios (%) that is the ratio of survival time (in days) for treated mice (T) to untreated control mice (C). The significance of the survival data of drug-treated groups *vs* untreated groups or of combined *vs* single therapy were evaluated by '*P*' values (student's *t*-test, unpaired data) (for further details, see Boesch *et al*., 1991*c*).

### Results

### *SDZ 280-446 selection*

SDZ 280-446 (Figure 1), the chemistry of which will be reported in a separate publication (G. Emmer *et al*., in preparation), was one of a series of semi-synthetic and non-immunosuppressive derivatives of SDZ 90-215, a natural cyclic peptolide isolated from a Fungus imperfecti (*Septoria sp*., Sandoz strain F/42508). It selection was performed through the same screening systems that led to the selection of SDZ PSC 833 (Gavériaux *et al*., 1991), using four pairs of Par- and MDR-tumour cell lines, searching for a broad '*in vitro* therapeutic window', i.e. the lowest possible intrinsic cytostatic activity together with the highest possible chemosensitising activity.

**Figure 1** Structure of SDZ 280-446. SDZ 280-446 is cyclo-[Pec$^{1}$ - MeVal$^{2}$ - Val$^{3}$ - (-O-t.Bu-MeAsp$^{4}$) - MeIle$^{5}$ - MeIle$^{6}$ - Gly$^{7}$ - MeVal$^{8}$ - (O-MeTyr$^{9}$) - L-Hpa$^{10}$]; empirical formula $C_{61}H_{99}N_{9}O_{14}$, Molecular Weight: 1182.6.

All MDR-cell lines were variants with high expression of Pgp/mdrl mRNA and indeed displaying a high level of MDR against several ACDs, particularly COL, VCR, VP-16 and DAU and/or DOX (Table I). The Par-P388 and Par-KB cell lines showed minimal IC50s for these agents, did not express detectable amounts of Pgp or mdrl mRNA and were not chemosensitisable by any of our RMAs known or suspected to work through inhibition of Pgp function. However the Par-CHO and Par-LoVo cell lines which expressed small but detectable amounts of Pgp or mdrl mRNA could be sensitised by micromolar concentrations of weak RMAs or by sub-micromolar concentrations of strong RMAs.

These four pairs of cell lines also showed variable sensitivity (IC50) to the RMAs (Table I). However there was no rule regarding the relative resistance of the Par- and MDR-cell variants for ACDs and for RMAs, nor regarding the intrinsic growth inhibition capacity of the RMAs. For instance, CsA was the most cytostatic RMA for Par-P388 cells and the least cytostatic RMA for MDR-KB cells, while SDZ PSC 833 was more cytostatic for MDR-KB cells than for Par-KB cells, that SDZ 280-446 IC50s were roughly similar for the growth inhibition of both sublines of CHO and LoVo cells, and so on. Though this is shown here for three RMAs and four cell line pairs only, our experience with several other RMAs and a few other cell lines does not suggest that there is a correlation between resistance to the ACD and resistance to the RMA, at least within the Pgp-mediated resistance context.

### In vitro *restoration of ACD-sensitivity: RM-strengths of SDZ 280-446, SDZ PSC 833 and CsA*

The sensitising capacity of the RMAs for various ACDs was studied as a function of the RMA concentration (for all three RMAs, $1\,\mu M = \pm 1.2\,\mu g\,ml^{-1}$ or $1\,\mu g\,ml = \pm 0.8\,\mu M$) and represented as isobolic curves (one per ACD and RMA combination). These isobolograms give the RMA concentrations on the X-axes and the ACD sensitivity gains on the Y-axes. Each gain is defined as the ratio of ACD [$IC50^{-}$/$IC50^{+}$] determined from ACD dose/response curves performed in absence ($IC50^{-}$) or presence ($IC50^{+}$) of RMA. Such isobolograms thus express the sensitising capacity of the RMAs for various ACDs as a function of the doses of RMAs.

The isobolograms shown in Figures 2–5 indicate that SDZ 280-446 (M.W. 1182.6) is about equipotent as RMA with SDZ PSC 833 (M.W. 1214.65) but much more potent than CsA (1206.6). Throughout all RMA, ACD and MDR-cell line combinations, it is apparent that equimolar concentrations of SDZ 280-446 and SDZ PSC 833 gave a stronger chemosensitisation (larger 'gains') than CsA; or, similarly, that equivalent chemosensitisation (similar 'gains') required lower concentrations of SDZ 280-446 and SDZ PSC 833 than of CsA. A simplified, yet less complete picture, is obtained by comparing the doses of RMA giving similar gains in sensitivity (e.g. a 10 fold gain, where achievable, as shown in Table II, which also includes verapamil (M.W. 491) for comparison).

As already found with SDZ PSC 833, a complete reversion of the resistance of the MDR cells (i.e. a gain in sensitivity equal to the relative resistance between the Par- and MDR-cell lines) could be obtained with the CHO, P388 and LoVo MDR cells, but not with the available variant of MDR-KB cells. The latter were however significantly sensitisable by SDZ 280-446, as well as by SDZ PSC 833 though not by CsA.

**Figure 2** Chemosensitisation of CHO cells *in vitro* by SDZ 280-446, SDZ PSC 833 and CsA. Par-cells (open symbols) and MDR-cells (closed symbols) were cultured with ranges of concentrations of colchicine, daunomycin, etoposide or vincristine together with a range of concentrations of SDZ 280-446 (M.W. 1182.6; ○ ●), SDZ PCS 833 (M.W. 1214.65; △ ▲) or CsA (M.W. 1206.6; □ ■) (X-axes). The ratios of ACD IC50s obtained in the absence of RMA and in the presence of each RMA concentration, i.e. the gains of sensitivity are recorded on the Y-axes. Each gain was determined from two individual ACD dose-cell growth inhibition curves at inactive or hyperactive RMA concentrations up to nine such determinations for mid-active RMA concentrations.

**Figure 3** Chemosensisation of KB cells *in vitro* by SDZ 280-446, SDZ PSC 833 and CsA. Same representation as in Figure 2.

Though the highest gains in sensitivity to ACDs were obtained with the MDR cells, significant gains were also found with cells from two Par-cell lines (CHO and LoVo). The latter Par-cells actually express small amounts of Pgp and thus are susceptible to chemosensitisation. On the contrary the two Par-cell lines which do not detectably express Pgp (Par-KB and Par-P388) were not chemosensitisable to any significant extent.

**Figure 4** Chemosensitisation of P388 cells *in vitro* by SDZ 280-446, SDZ PSC 833 and CsA. Same representation as in Figure 2.

**Figure 5** Chemosensisation of LoVo cells *in vitro* by SDZ 280-446, SDZ PSC 833 and CsA. Same representation as in Figure 2.

Particularly with the most potent RMAs, the chemosensitising activity was quite distinct from the negligible growth inhibitory effects of the RMAs themselves. For example, with MDR-P388 cells, 10-fold gains were obtained with less than 0.1 μg ml$^{-1}$ (less than 0.08 μM) SDZ PSC 833 or SDZ 280-446 (Table II), while the IC50s for these RMAs were 6–7 μg ml$^{-1}$ (Table I). Similarly with MDR-LoVo cells, about 0.2 μM SDZ PSC 833 or 0.4 μM SDZ 280-446 gave 10-fold gains (Table II), while the IC50s of both RMAs reached about 15 μM (13.5–14.5 μg ml$^{-1}$) (Table I).

**Table II** RMA molarities giving either a 10-fold GAIN ( = degree of chemosensitisation) or the maximal achievable GAIN of sensitivity

| | | *Verapamil* | | *CsA* | | *SDZ PSC 833* | | *SDZ 280-446* | |
|---|---|---|---|---|---|---|---|---|---|
| *Cell lines* | *ACD* | *Gain* | *μM* | *Gain* | *μM* | *Gain* | *μM* | *Gain* | *μM* |
| Par-CHO | COL | 9.1 | 50 | 8.9 | 1.67 | 10 | 0.29 | 9.5 | 4.2 |
| | VCR | 10 | 5.7 | 10 | 0.63 | 10 | 0.023 | 10 | 0.091 |
| | DAU | 10 | 34 | 7.4 | 4.2 | 10 | 0.39 | 8.1 | 4.2 |
| | VP-16 | 4.8 | 30 | 8.3 | 2.5 | 7.1 | 0.82 | 6.7 | 4.2 |
| MDR-CHO | COL | 10 | 6.4 | 10 | 0.37 | 10 | 0.017 | 10 | 0.013 |
| | VCR | 10 | 2.8 | 10 | 0.50 | 10 | 0.025 | 10 | 0.013 |
| | DAU | 10 | 1.5 | 10 | 0.35 | 10 | 0.011 | 10 | 0.009 |
| | VP-16 | 10 | 12.3 | 10 | 0.50 | 10 | 0.017 | 10 | 0.016 |
| Par-LoVo | COL | ND | | 3.7 | 8.3 | 4.8 | 2.5 | 4.9 | 2.5 |
| | VCR | ND | | 3.7 | 8.3 | 10 | 0.034 | 10 | 0.12 |
| | DAU | ND | | 4.3 | 2.5 | 3.8 | 1.6 | 4.3 | 4.2 |
| | VP-16 | ND | | 4.7 | 2.5 | 2.7 | 1.6 | 2.8 | 2.5 |
| MDR-LoVo | COL | ND | | 10 | 2.68 | 10 | 0.21 | 10 | 0.28 |
| | VCR | ND | | 10 | 1.0 | 10 | 0.26 | 10 | 0.38 |
| | DAU | ND | | 10 | 1.77 | 10 | 0.15 | 10 | 0.57 |
| | VP-16 | ND | | 10 | 2.50 | 10 | 0.25 | 10 | 0.44 |
| MDR-P388 | COL | 10 | 11.7 | 10 | 0.32 | 10 | 0.070 | 10 | 0.061 |
| | VCR | 10 | 3.1 | 10 | 0.27 | 10 | 0.033 | 10 | 0.040 |
| | DAU | 10 | 3.3 | 10 | 0.30 | 10 | 0.045 | 10 | 0.068 |
| | DOX | 10 | 3.9 | 10 | 0.3 | 10 | 0.034 | 10 | 0.059 |
| | VP-16 | 10 | 11.4 | 10 | 0.21 | 10 | 0.026 | 10 | 0.076 |
| MDR-KB | COL | 10 | 11.8 | 1.9 | 2.5 | 10 | 0.86 | 10 | 0.57 |
| | VCR | 10 | 3.1 | 1.7 | 4.2 | 10 | 0.86 | 10 | 0.71 |
| | DAU | 10 | 10.5 | 4.7 | 4.2 | 10 | 0.84 | 10 | 0.83 |
| | VP-16 | 3.5 | 30 | 1.1 | 4.2 | 10 | 1.89 | 10 | 0.93 |

*Restoration of DAU retention in MDR-P388 cells by CsA, SDZ PSC 833 and SDZ 280-446*

The intracellular ACD retention was measured by the degree of anthracycline-fluorescence of the P388 cells of both Par- and MDR-lines following *in vitro* exposure to DAU, either with the fluorescence-microscope (end point active concentration) or with a FASC (more quantitative data). While Par-P388 cells displayed a high nuclear fluorescence upon *in vitro* exposure to DAU, and MDR-P388 cells did not fluoresce at all, indicating the low intracellular DAU content. In fluorescence studies, the bright nuclear fluorescence of Par-P388 cells was identical whether they had been exposed to the RMA or not. In contrast, there was a RMA dose-dependent increase in the degree of nuclear fluorescence of the MDR-P388 cells. In order to reach a similar fluorescence intensity (almost as bright as the fluorescence displayed by the Par-P388 cells) the MDR-P388 cells had to be exposed to 10–30 $\mu g\ ml^{-1}$ CsA, 1–3 $\mu g\ ml^{-1}$ SDZ 280-446 or 0.3–1.0 $\mu g\ ml^{-1}$ SDZ PSC 833. Both SDZ PSC 833 and SDZ 280-446 at 30 $\mu g\ ml^{-1}$ allowed a complete restoration of the DAU retention by the MDR-P388 cells, whereas CsA could not (not shown).

Though they did not allow distinction of the 'specific', i.e. nuclear, fluorescence from the whole cell fluorescence, flow cytometry analyses of DAU retention in Par-P388 and MDR-P388 cells provided less subjective comparison of the DAU retention in the two cell lines as a function of the RMA concentration. In the absence of RMA treatment, the MDR-P388 cells displayed low fluorescence levels corresponding to 4.5–5.8% of the Par-P388 cell fluorescence levels. Definite shifts of the fluorescence profiles of the RMA-treated MDR-P388 cell populations were observed at low concentrations of all three RMAs. As already described for several other RMAs (Boesch *et al.*, 1991*b*), these shifts were obtained at different concentrations and showed subtle differences for the different RMAs.

In order to facilitate the comparison of the effects of the RMAs on the fluorescence levels of Par-P388 and MDR-P388 cells, the peak fluorescence levels (Y-axes) were plotted *vs* the RMA concentrations (X-axes) in Figure 6 which compares SDZ PSC 833 and SDZ 280-446 with CsA. The lowest RMA concentrations sufficient to restore *fully* DAU retention in MDR-P388 cells were 30 $\mu g\ ml^{-1}$ for CsA, 1–3 $\mu g\ ml^{-1}$ for SDZ PSC 833 and 3–10 $\mu g\ ml^{-1}$ for SDZ 280-446. Therefore, by measuring the short term, RMA-mediated inhibition of Pgp function, SDZ PSC 833 was found to be about three times more active than SDZ 280-446 and 10–30 times more active than CsA.

In vivo *efficacy of SDZ 280-446*

*Vinca alkaloids (VCR or VBL) as ACD* Although they could prolong the survival of mice bearing the Par-P388 tumour cells, neither VCR nor VBL alone could significantly prolong the survival of MDR-P388 cell-grafted mice up to the high ACD dosages where they became severely toxic for the mice themselves (i.e. toxic for tumour-free mice) (Boesch *et al.*, 1991*c*).

Four independent experiments using vinca alkaloids allowed to evaluate the chemosensitising efficacy of SDZ 280-446 (Table III). In all four experiments DBA/2 mice were grafted at Day 0 with five millions (parts A and B) or one million (parts C and D) Par-P388 or MDR-P388 tumour cells. Then, the RMA or its placebo was given by gavage per os and the vinca alkaloid was injected i.p. for 5 consecutive days.

The vinca alkaloids alone in the 15–100 $\mu g\ kg^{-1}$ daily dosage range could not significantly prolong the survival of the mice bearing a MDR-tumour and were variably effective for mice grafted with Par-tumour cells. The daily treatment with 100 $mg\ kg^{-1}$ of SDZ 280-446 alone had also no significant effects on the survival of tumour-bearing mice. In contrast the vinca alkaloids could significantly increase the survival of the MDR-tumour-bearing mice when administered i.p. together with SDZ 280-446 per os. The effect of SDZ 280-446 was dose-dependent, the 100 $mg\ kg^{-1}$ daily dosage appearing to be most effective for this treatment protocol. Curiously enough mice bearing Par-tumour and treated by the combined therapy could also show increased survival in comparison with mice treated with vinca alkaloid alone. Though this may be accounted for by an increased bioavailability of ACD when used in combination with SDZ 280-446, it would not suffice to explain the higher efficacy of vinca alkaloids on the MDR-tumour: as mentioned earlier it was not possible to interfere with MDR-tumour growth-mediated mouse death by increasing the daily dosage of vinca alkaloids up to the doses which were severely toxic for tumour-free mice.

**Figure 6** Effect of the RMAs on the DAU-retention by Par-P388 (O) and MDR-P388 (●) cells. SDZ PSC 833 and SDZ 280-446 were compared with CsA ("SIM"). The cells were incubated for 30 min at 37°C with 20 μM DAU and a range of RMA concentrations. After washing the cells were incubated in DAU- or RMA-free medium at 37°C. The fluorescence measured by flow cytometry was represented on a logarithmic scale. The peak fluorescence levels (Y-axes) are shown *vs* the RMA concentrations (X-axes).

*Topoisomerase II inhibitor (DOX) as ACD*

Previous experiments indicated that treatment with DOX i.p. three times at 4 day intervals starting on day 1 after tumour inoculation achieved a significant prolongation of survival of Par-P388 tumour-bearing mice, but not of MDR-tumour-bearing mice. However a pre-treatment with SDZ PSC 833 24 h before each DOX administration (2 mg kg$^{-1}$ i.p.) could clearly increase the survival of MDR-P388 tumour-bearing mice (T/C of about 170%). When SDZ PSC 833 pharmacokinetics could be taken into account, the time between SDZ PSC 833 p.o. and DOX i.p. was then reduced to 4 h and the SDZ PSC 833 dose was reduced to 50 or 25 mg kg$^{-1}$; this dramatically improved the efficacy of the combined chemotherapy (Boesch *et al.*, 1991*c*).

Though pharmacokinetic information on SDZ 280-446 was lacking, a similar treatment protocol was assayed. Thus the tumour cells were injected i.p. at day 0 (4 h before 0 time), SDZ 280-446 or its vehicle was given p.o. at days 0, 4 and 8, and DOX or its vehicle was injected i.p. 4 h after each gavage with the RMA. The data of one complete experiment are shown in Table IV. As expected, the treatment with DOX alone did not result in increased mouse survival (MST of 13.6 instead of 12.8 days). Similarly, no remarkable effects on mouse survival were observed in the groups treated with SDZ 280-446 alone. However, a highly significant prolongation of the survival of the MDR-P388 tumour-bearing mice was obtained by the combined therapy: with SDZ 280-446 (at 25 and 50 mg kg$^{-1}$ given 4 h before each DOX treatment), the mouse survival reached about 31 days, the combined therapy being thus roughly 2–3 fold more effective than the single DOX therapy.

### Discussion

The ability of the cyclic peptolide SDZ 280-446 to sensitise *in vitro* tumour cells whose resistance is due to Pgp-mediated ACD efflux has been well documented for four different pairs of Par- and MDR-cell lines, from three different species

**Table III** *In vivo* chemosensitising activity of SDZ 280-446 for vinca alkaloids in Par-P388 and MDR-P388 tumour-bearing DBA/2 mice

| *Chemotherapy SDZ 280-446 p.o. (mg kg$^{-1}$)* | *ACD i.p. (μg kg$^{-1}$)* | *Parental-P388 tumour MST±IV days* | *(n)* | *'P'* | *T/C (%)* | *MDR-P388 tumour MST±IV days* | *(n)* | *'P'* | *T/C (%)* |
|---|---|---|---|---|---|---|---|---|---|
| *Part A*[a] | | | | | | | | | |
| V | V | 22.4±0.5 | (9) | | 100 | 8.1±1.3 | (9) | | 100 |
| V | VCR, 100 | 22.0±5.1 | (8) | 0.8 | 98 | 8.4±1.2 | (9) | 0.58 | 104 |
| 100 | V | 24.2±2.8 | (6) | 0.089 | 108 | 7.8±0.5 | (5) | 0.61 | 96 |
| 100 | VCR, 100 | 35.5±9.7 | (4) | 0.001 | 158 | 12.5±1.8 | (10) | <0.001 | 154 |
| *Part B*[a] | | | | | | | | | |
| V | V | 20.3±0.5 | (7) | | 100 | 7.7±1.5 | (7) | | 100 |
| V | VBL, 100 | 27.1±1.8 | (7) | <0.001 | 133 | 8.4±1.0 | (7) | 0.31 | 109 |
| 100 | V | 20.2±0.4 | (5) | 0.76 | 100 | 7.0±0.0 | (5) | 0.32 | 91 |
| 100 | VBL, 30 | 30.1±2.3 | (7) | <0.001 | 148 | 24.5±3.9 | (8) | <0.001 | 318 |
| *Part C*[a] | | | | | | | | | |
| V | V | 21.0±3.8 | (8) | | 100 | 10.7±1.4 | (8) | | 100 |
| V | VBL, 100 | 25.8±7.4 | (8) | 0.13 | 123 | 11.4±1.6 | (8) | 0.42 | 107 |
| 100 | V | 22.6±1.3 | (8) | 0.27 | 108 | 10.3±1.5 | (6) | 0.61 | 96 |
| 100 | VBL, 30 | 35.9±6.2 | (7) | <0.001 | 171 | 19.6±5.6 | (9) | 0.001 | 183 |
| 50 | VBL, 30 | N.D. | | | | 12.5±1.5 | (10) | 0.021 | 117 |
| 25 | VBL, 30 | N.D. | | | | 11.2±1.0 | (9) | 0.44 | 105 |
| 12 | VBL, 30 | N.D. | | | | 10.0±1.3 | (10) | 0.27 | 93 |
| *Part D*[a] | | | | | | | | | |
| V | V | 21.6±1.6 | (10) | | 100 | 10.4±1.6 | (10) | | 100 |
| V | VBL, 100 | 26.5±3.6 | (6) | 0.019 | 123 | 9.1±0.4 | (7) | 0.042 | 87 |
| V | VBL, 30 | 21.6±1.9 | (8) | 1.0 | 100 | N.D. | | | |
| 100 | V | 23.5±4.0 | (6) | 0.31 | 109 | 9.5±0.4 | (6) | 0.45 | 91 |
| 100 | VBL, 30 | 28.0±4.1 | (6) | 0.011 | 130 | 16.6±2.7 | (8) | 0.001 | 160 |
| 100 | VBL, 15 | N.D. | | | | 15.9±3.1 | (8) | 0.001 | 153 |

[a]Parts A, B, C & D represent independent experiments. Grafted tumour cells: 5 × $10^6$ (parts A & B) or $10^6$ cells (parts C & D). Drug(s) administered at days 0, 2 & 4 (part A) or at days 0, 1, 2, 3 & 4 (parts B, C & D). V = vehicle, (*n*) = number of mice.

**Table IV** *In vivo* activity of three cycles of SDZ 280-446 p.o. 4 h before DOX i.p. at 4 day intervals for MDR-P388 tumour-bearing B6D2F1 mice

| *Chemotherapy* | | *MDR-P388 tumour* | | |
|---|---|---|---|---|
| *SDZ 280-446*[a] *p.o. (mg kg*$^{-1}$*)* | *DOX i.p. (mg kg*$^{-1}$*)* | *MST±IV days* | *'P'* | *T/C (%)* |
| V | V | 12.8± 2.1 | 0.074 | 100 |
| V | 2 | 13.6± 0.9 | 0.006 | 106 |
| 25 | V | 12.8± 1.3 | 0.140 | 100 |
| 50 | V | 14.2± 1.2 | 0.002 | 111 |
| 25 | 2 | 31.1±11.9 | 0.002 | 243 |
| 50 | 2 | 31.3± 5.1 | <0.001 | 245 |

[a]SDZ 280-446 was administered per os 4 h before DOX to MDR-P388 tumour-bearing B6D2F1 mice (5–8 mice per group); V = vehicle.

(including two human cell line pairs) representing four different cell lineages, and using four different ACD classes. It is clear that SDZ 280-446 and the cyclosporin derivative SDZ PSC 833 (Gavériaux *et al.*, 1991; Boesch *et al.*, 1991*c*) achieve the same high level of chemosensitisation which is about one order of magnitude more active than CsA, which is itself about one order of magnitude higher than other known chemosensitisers ([Boesch *et al.*, 1991*a*] including verapamil, quinidine and amiodarone which have already entered clinical trials in MDR reversal).

DAU retention could be restored in MDR-P388 cells by chemosensitisers which bind to the Pgp pump, such as Ver and CsA. SDZ PSC 833 could restore this retention at lower doses than CsA which was itself more active than amiodarone, verapamil, quinidine and all other tested RMAs (Boesch *et al.*, 1991*b*). SDZ 280-446 was also identified as one of the most potent RMA by this assay. The lowest RMA concentrations sufficient to fully restore DAU retention in MDR-P388 cells were 30 μg ml$^{-1}$ for CsA, 1–3 μg ml$^{-1}$ for SDZ PSC 833 and 3–10 μg ml$^{-1}$ for SDZ 280-446. Therefore, by the assay which measures the short term inhibition of Pgp function by RMA-pretreatment of the MDR-P388 cells, we could evaluate that SDZ PSC 833 was about three times better than SDZ 280-446 and 10–30 times better than CsA. In the DAU-growth inhibition assay a 6-fold factor only was found for the concentrations of CsA (0.30 μM) and SDZ PSC 833 (0.045 μM) giving a gain of 10 (Table II); yet in that assay, SDZ PSC 833 and SDZ 280-446 (0.068 μM) had more similar potencies.

SDZ 280-446 was also effective as a chemosensitiser when given orally *in vivo*. In a syngeneic mouse model (P388 tumour cells of DBA/2 origin growing on DBA/2 mice), combined therapy with vinca alkaloids given i.p. and SDZ 280-446 given per os for 5 consecutive days significantly prolonged the survival of tumour-bearing mice, when compared with mice receiving vinca alkaloids alone.

This clearly beneficial effect of the combined therapy was seen principally in recipients of MDR-tumour cells, and not or only moderately in recipients of Par-tumour cells which lack resistance. This was similar to what was observed with SDZ PSC 833 (Boesch *et al.*, 1991*c*). In the latter case the effect of the combined therapy on the Par-tumour bearing recipients could be explained by an increased bioavailability of the ACD through alterations of the P450 system, as typical for cyclosporins. Such pharmacokinetic interactions also occur in the case of SDZ 280-446, but presently available data are still too fragmentary to allow a comparison with SDZ PSC 833. Whichever the increased ACD-bioavailability which is beneficial for Par-tumour bearing mice, it would not be sufficient for the MDR-tumour bearing mice since it was impossible to obtain any significant prolongation of survival of MDR-P388 tumour-bearing mice by increasing the daily dosage of vinca alkaloids (Boesch *et al.*, 1991*c*). This clearly shows that the beneficial effect of combining SDZ 280-446 with a low dose of vinca alkaloid comes essentially from a direct neutralisation of the Pgp which is overexpressed on MDR-tumour cells by the cyclic peptolide, rather than indirectly through altering ACD bioavailability.

Although only few treatment protocols with vinca alkaloids have been tested so far, clear increases in survival times were regularly obtained, leaving hope for possible improvement of the therapy, using other dosing schedule. Particularly our results using SDZ 280-446 orally compare well with the best results reported for a nifedipine analog (AHC-52) given i.p. with the tumour (Tsuruo *et al.*, 1981; Shinoda *et al.*, 1989). The superiority of SDZ 280-446 may even be higher than appears from a simple comparison of the survival increases achieved by SDZ 280-446 and AHC-52 as RMAs. Indeed in the AHC-52 study two different MDR-P388 cell lines were used: a P388/VCR which had only a moderate resistance (12-fold) and a P388/ADR cell line which had a high resistance (150-fold), in comparison with the Par-P388 line. Combined therapy by AHC-52 and vinca alkaloids had remarkable effects on the P388/VCR tumour-bearing mice but showed no efficacy or only marginal effects with mice bearing the high resistant P388/ADR tumours. Since our DOX-driven MDR-P388 cell line was actually as highly resistant as the aforementioned P388/ADR line, SDZ 280-446 must be much more potent than AHC-52 *in vivo*.

All presently available data on the *in vivo* efficacy of SDZ 280-446 have been obtained by reproducing early SDZ PSC 833-treatment protocols, where the mice were exposed to a 25 times higher cumulative SDZ PSC 833 dosage than in our later experiments which nevertheless showed much higher efficacy. It is definite that further improvements of the efficacy of the SDZ 280-446-aided chemotherapy will be possible when a better knowledge of its *in vivo* pharmacology is available.

Due to the large differences in the pharmacokinetics and metabolism of MDR-reversing agents and cytostatics in mice and man, the optimal therapeutic schedule will clearly have to be established directly on patients, bearing in mind the possibility of enhanced toxicity with the combination. However it is highly probable that this would come from the enhanced bioavailability and effects of the ACD rather than of intrinsic SDZ 280-446 toxicity, since studies in rats have shown that SDZ 280-446 is virtually devoid of toxicity when given orally at doses of 500 mg kg$^{-1}$ or more for 2 weeks (P. Donatsch, Sandoz Toxicology Dept, unpublished data).

Based on the results obtained *in vivo* it can be anticipated that SDZ 280-446 given orally shortly prior to and during cycles of cytostatic therapy could be effective in reversing resistance of tumours exhibiting MDR to chemotherapy in a wide variety of patients. Although 25–100 mg kg$^{-1}$ of SDZ 280-446 seems to be required in the present protocols to see a beneficial effect of ACD therapy on the MDR-tumour bearing mice, all naturally occurring human MDR cancers are far less resistant than our MDR-tumour cell line models. Our MDR-P388 cell line is about 100–150 fold more resistant than the Par-P388 cell line. Although it is difficult to extrapolate from mdr1 mRNA levels or Pgp protein levels to actual resistance degrees, it does not seem that 'naturally-occurring' MDR cancers will ever show resistance indices higher than 5–10 (see e.g. Kanamaru *et al.*, 1989). This, together with the generally higher metabolism rate observed in small animals, means that our *in vivo* assays might overestimate by an order of magnitude the dosages of RMAs, such as SDZ 280-446 and SDZ PSC 833, which will be required in clinical practice.

The mechanisms of action of SDZ PSC 833 and SDZ 280-446 are currently being compared as well as their suitability for *in vivo* chemosensitisation of MDR-tumour. Ideally, the RMA should share three properties: (1) the highest intrinsic RM-activity to allow a complete blockade of Pgp at low RMA dosages; (2) the largest therapeutic window of ACD dosages, thus the highest flexibility of chemotherapy protocols; and (3) the broadest spectrum of activity towards most Pgp-mediated tumour types. Whether SDZ PSC 833 might be better in some cases, and SDZ 280-446 in some other remains an open question.

The authors are very grateful to Drs Maria Grandi, Victor Ling and Ira Pastan who provided the cell lines used in this study, Kurt Mueller for help with the FACS analyses, Hans Fliri and Trevor

Payne for discussions and for their help in the selection of SDZ 280-446, Peter Hiestand for confirming its lack of immunosuppressive activity, Peter Donatsch for communicating us his preliminary data on bioavailability and toxicology of SDZ 280-446.

### References

BELLAMY, W.T., DALTON, W.S. & DORR, R.T. (1990). The clinical relevance of multidrug resistance. *Cancer Invest.*, **8**, 547.

BOESCH, D., GAVÉRIAUX, C. & LOOR, F. (1991*a*). Reversal of multidrug-resistance in CHO cells by cyclosporin A and other resistance modifying agents. *J. Cell. Pharmacol.*, **2**, 92.

BOESCH, D., MULLER, K., POURTIER-MANZANEDO, A. & LOOR, F. (1991*b*). Restoration of daunomycin retention in highly resistant P-glycoprotein-expressing P388 cells by submicromolar concentrations of SDZ PSC-833, a non-immunosuppressive cyclosporin derivative. *Exp. Cell. Res.*, **196**, 26.

BOESCH, D., GAVÉRIAUX, C., JACHEZ, B., POURTIER-MANZANEDO, A., BOLLINGER, P. & LOOR, F. (1991*c*) SDZ PSC 833: *in vivo* circumvention of P-glycoprotein-mediated multidrug-resistance of tumor cells. *Cancer Res.*, **51**, 4226.

BRADLEY, G., JURANKA, P.F. & LING, V. (1988). Mechanism of multidrug resistance. *Biochim. Biophys. Acta*, **948**, 87.

ENDICOTT, J.A. & LING, V. (1989). The biochemistry of P-glycoprotein-mediated multidrug resistance. *Annu. Rev. Biochem.*, **58**, 137.

FORD, J.M., PROZIALECK, W.C. & HAIT, W.N. (1989). Structural features determining activity of phenothiazines and related drugs for inhibition of cell growth and reversal of multidrug resistance. *Mol. Pharmacol.*, **35**, 105.

FOXWELL, B.M.J., MACKIE, A., LING, V. & RYFFEL, B. (1989). Identification of the multidrug resistance related P-glycoprotein as a cyclosporine binding protein. *Mol. Pharmacol.*, **36**, 543.

GAVÉRIAUX, C., BOESCH, D., BOELSTERLI, J.J. & 6 others (1989). Overcoming multidrug resistance in Chinese hamster ovary cells *in vitro* by cyclosporin A and non-immunosuppressive derivatives. *Br. J. Cancer*, **60**, 867.

GAVÉRIAUX, C., BOESCH, D., JACHEZ, B., BOLLINGER, P., PAYNE, T. & LOOR, F. (1991). SDZ PSC 833, a non-immunosuppressive cyclosporin analog, is a very potent multidrug-resistance modifyer. *J. Cell. Pharmacol.*, **2**, 225.

GEORGES, E., SHAROM, F.J. & LING, V. (1990). Multidrug resistance and chemosensitization: therapeutic implications for cancer chemotherapy. *Adv. Pharmacol.*, **21**, 185.

HOFSI, E. & NISSEN-MEYER, J. (1990). Reversal of multidrug resistance by lipophilic drugs. *Cancer Res.*, **50**, 3997.

JURANKA, P.F., ZASTAWNY, R.L. & LING, V. (1989). P-glycoprotein: multidrug-resistance and a superfamily of membrane-associated transport proteins. *FASEB J.*, **3**, 2583.

KALLMAN, R.F. (1987). *Rodent Tumor Models in Experimental Cancer Therapy*, Pergamon Press.

KANAMARU, H., KAKEHI, Y., YOSHIDA, O., NAHANISHI, S., PASTAN, I. & GOTTESMAN, M.M. (1989). MDR1 RNA levels in human renal cell carcinomas: correlation with grade and prediction of reversal of doxorubic in resistance by quinidine in tumor explants. *J. Natl Cancer Inst.*, **81**, 844.

MOSCOW, J.A. & COWAN, K.H. (1990). Multidrug resistance. Cancer Chemotherapy and *Biol. Response Modifyers Ann.*, **11**, 97.

SHINODA, H., INABA, M. & TSURUO, T. (1989). *In vivo* circumvention of vincristine resistance in mice with P388 leukemia using a novel compound, AHC-52. *Cancer Res.*, **49**, 1722.

TAKAHASHI, N., HAYANO, T. & SUZUKI, M. (1989). Peptidyl-prolyl cis-trans isomerase is the cyclosporin A-binding protein cyclophilin. *Nature*, **337**, 473.

TSURUO, T., IIDA, H., TSUKAGOSHI, S. & SAKURAI, Y. (1981). Overcoming of vincristine resistance in P388 leukemia *in vivo* and *in vitro* through enhanced cytotoxicity of vincristine and vinblastine by verapamil. *Cancer Res.*, **41**, 1967.

TWENTYMAN, P.R. (1988). A possible role for cyclosporins in cancer chemotherapy. *Anticancer Res.*, **8**, 985.

ZAMORA, J.M., PEARCE, H.L. & BECK, W.T. (1988). Physical-chemical properties shared by compounds that modulate multidrug resistance in human leukemic cells. *Mol. Pharmacol.*, **33**, 454.